# DeepHipp: accurate segmentation of hippocampus using 3D dense-block based on attention mechanism

**DOI:** 10.1186/s12880-023-01103-5

**Published:** 2023-10-13

**Authors:** Han Wang, Cai Lei, Di Zhao, Liwei Gao, Jingyang Gao

**Affiliations:** 1 Department of Information Science and Technology, Beijing University of Chemical Technology, Beijing, China; 2 Institute of Computing Technology, Chinese Academy of Sciences, Beijing, China; 3 Department of Radiation Oncology China, Japan Friendship Hospital, Beijing, China

**Keywords:** Segmentation of hippocampus, Deep learning, Dense block, Attention, Data augmentation

## Abstract

**Background:**

The hippocampus is a key area of the brain responsible for learning, memory, and other abilities. Accurately segmenting the hippocampus and precisely calculating the volume of the hippocampus is of great significance for predicting Alzheimer’s disease and amnesia. Most of the segmentation algorithms currently involved are based on templates, such as the more popular FreeSufer.

**Methods:**

This study proposes Deephipp, a deep learning network based on a 3D dense block using an attention mechanism for accurate segmentation of the hippocampus. DeepHipp is based on the following novelties: (i) DeepHipp adopts powerful data augmentation schemes to enhance the segmentation ability. (ii) DeepHipp is designed to incorporate 3D dense-block to capture multiple-scale features of the hippocampus. (iii) DeepHipp creatively uses the attention mechanism in the field of hippocampal image segmentation, extracting useful hippocampus information in a massive feature map, and improving the accuracy and sensitivity of the model.

**Conclusions:**

We describe the illustrative results and show extensive qualitative and quantitative comparisons with other methods. Our achievement demonstrates that the accuracy of DeepHipp can reach 83.63%, which is superior to most existing methods in terms of accuracy and efficiency of hippocampus segmentation. It is noticeable that deep learning can potentially lead to an effective segmentation of medical images.

## Introduction

Magnetic resonance imaging (MRI) can reveal the structural characteristics of various brain regions. As an important part of the brain, the hippocampus plays a very important role in the triggering mechanism of related diseases such as the nervous system. Many diseases are related to the hippocampus. For example: Alzheimer’s disease [1], PTSD [2], schizophrenia [3], obsessive-compulsive disorder [4], depression [5], dementia [6], and even autism [7]. To use neuroimaging to assess disease progression and the effectiveness of treatment strategies, high-precision, repeatable measurement assessments of hippocampal structures are required. Dill et al. [8] have reviewed the evolution and the state of the art of automated methods for hippocampus segmentation in MRI, which can be divided into four stages: thresholding and region growth method, shape models, machine learning, and region learning. With the development of this field, many automated methods and tools have been developed. Chupin et al. [9] developed a method for automatic hippocampus segmentation, and they used the obtained hippocampus volumes to automatically discriminate AD patients, MCI patients, and elderly controls, with an accuracy of over 70%.

At present, there are a lot of tools for qualitative and quantitative analysis of the hippocampus, such as Freesurfer [10], ANTs [11], FSL [12], etc. However, most of them are based on template registration with limited scope, and these tools take a long time to detect and consume a lot of manpower and resources. Among these tools, FreeSurfer is one of the most widely used and representative tools. Freesurfer has been highly praised by the industry since its birth. It is known for its high-precision segmentation of brain regions and is the preferred tool in many areas of brain analysis [13]. However, Freesurfer has a disadvantage in that it takes too long to run a project. At the same time, the Freesurfer tool is resource-intensive. Running Freesurfer requires a large number of computing resources.

With the development of deep learning in the field of medical imaging, many disease prediction, imaging diagnosis, and pathological analysis problems have been solved with deep learning techniques, such as cardiac diagnosis [14], kidney diagnosis [15], and brain functional structure analysis [16]. In recent years, the most prominent application areas are fundus detection [17], lung nodule detection [18], and gastric cancer pathology [19]. Deep learning has made remarkable achievements. At the same time, segmentation algorithms based on deep learning have emerged in recent years. For example, Havaei [20] proposed a multiscale feature fusion segmentation network. Kamnitsas [21] first proposed the concept of 3D convolution for fully connected multiscale CNN (Convolution Neural Network). Kayalibay [22] invented the Unet architecture and achieved great success in Brain Tumor Segmentation with Deep Neural Networks (BraTS) in 2015. Notably, they employed a Jaccard loss function that intrinsically handles class imbalances. They make use of the large receptive field of their architecture to process entire patients at once. With the increasing depth of the neural network, there are also some problems. For a network with very deep layers, the vanishing gradient problem easily occurs. For example, in the 2016 ImageNet [23] Competition, Shangtang Technology achieved 1207 layers of a network. At the same time, an excessively large network would cause parameters explosion, which makes training difficult to converge, such as the Sparsely-Gated Mixture-of-Experts layer, MoE [24]. MoE contains thousands of sub-networks, and each network has as many as 137 billion parameters. Further, with Google’s Attention is All You Need [25] proposed, the industry began to put more emphasis on the application of attention models in natural language processing and computer vision. As for hippocampal segmentation, Manjon et al. [26] modified the structure of Unet and invented DS-UNet3D for automatic hippocampus subfield segmentation that they called DeepHIPS in 2022.

This paper proposes a deep neural network using T1 data to segment the bilateral hippocampus called DeepHipp. DeepHipp no longer uses traditional competition datasets as experimental data but instead uses real ADNI [27] datasets. We hope to verify the practicality and precision of DeepHipp in clinical testing through our method. Since the ADNI data does not have gold-standard manually-tagged labels, we use FreeSurfer to process the raw data and get the hippocampus masks. The hippocampus segmented by FreeSurfer is a standard form in terms of shape and volume density. Such data is not very robust to train the deep learning models. To make the model have better anatomical variability and MRI sequence variability, DeepHipp uses a powerful data augmentation scheme, including cropping, scaling, and nonlinear geometric transformation. With a powerful data amplification solution, DeepHipp can learn more useful information, enabling feature maps in the network to capture more details. In this paper, DeepHipp adopts a data augmentation scheme and integrates 3D dense-block into the DeepHipp to achieve a more accurate segmentation model. The dense block reuses features through the connection on the channels. Furthermore, because medical imaging has three-dimensional characteristics, our 3D module can fit the data very well. Dense-block is a more radical connection mechanism: it connects all feature maps, specifically, each layer accepts all the previous layers as its additional input. Dense-block can directly concatenate feature maps from different layers, which can achieve the combination of image information. Moreover, for the large number of feature maps generated by the Dense-block, we hope that DeepHipp can capture useful information in the massive feature maps. Therefore, we adopted the attention mechanism that improves the sensitivity and accuracy of target region prediction. It is noticeable attention mechanism can not only improve the prediction accuracy but also eliminate the influence of the irrelevant areas, which is equivalent to increasing the depth of the network without increasing the number of layers.

The attention mechanism is used for feature maps from different levels so that the attention mechanism can focus on the hippocampus region of interest, and automatically learn the valuable semantic information. In particular, the training loss is guided by the attention map, and only the loss in the hippocampus position is back forward. This corresponds to letting the shallow layers of the network identify the hippocampus outline, while the deep layers pay attention to the texture of the hippocampus.

## Methods

DeepHipp is a hippocampus segmentation tool based on deep learning development. It integrates the latest attention mechanism into the hippocampal target segmentation, which improves the ability of model segmentation. Meanwhile, DeepHipp incorporates the dense-block residual module in each layer of the network, which avoids the disappearance of the gradient. The convolutions used by DeepHipp are all 3D, which is a good fit for three-dimensional medical images. DeepHipp uses a powerful data augmentation mechanism, not only the number of amplifications in the original data set but also geometric transformation, voxel points density, and spatial coordinate transition. We introduce each aspect of DeepHipp in the following.

### Data preprocessing

For initial registration, we use FSL to process ADNI data uniformly and normalize the data into standard space to prepare for subsequent operations. Since we do not have gold-standard manually-tagged masks, we use the results of FreeSurfer segmentation as the training label for DeepHipp. FreeSurfer has a long history as a recognized brain segmentation tool in the industry and can achieve high accuracy in hippocampus segmentation. The reliability of Freesurfer has been proven in many ways, for example, Brown et al. proved the result of Freesurfer is robust [28]. Because we use the 3D network, the scale of network parameters is much larger than a 2D network, so we need to normalize the data. We do data preprocessing from the following three aspects.

#### Quantity expansion

To obtain a larger number of training data sets based on the original data, we need to increase the original data. First, we used a histogram equalization technique for all data to enhance the image contrast. Using histogram equalization to reduce the image chromatic aberration. It makes the picture look more natural and comfortable. Secondly, we use the technique of random rotation on the original image. It can also amplify the data amount. We perform a random angular rotation of the 3D data so that the original data is presented at different angles, which helps to enhance the robustness of the DeepHipp.

#### Geometric augmentation

We know that the parameters of the 3D convolution network are exponential times of the 2D convolutional network parameters. Moreover, because the medical data often has a large number of bytes of a single image, this greatly limits the batch-size settings and is also a great challenge for the GPU’s memory. To allow the network to accommodate more batch size, we copied three copies of the data processed in 2.1.1. In the first data, we cut the blank area of the original brain data based on the blank edges shown in the NII file and only retain the useful brain area. In the second data, we resize and normalize the brain as a dense whole, which reduces the distance between the voxel and enables the network to learn at different scales. In the third copy, we resample each individual, which randomly distorts the entire brain region, such as stretching or compressing in a certain direction. Moreover, the distorted images are rescaled into the standard size, which can be accommodated by the network.

#### Detailed explanation of operation methods

**Fig. 1:**
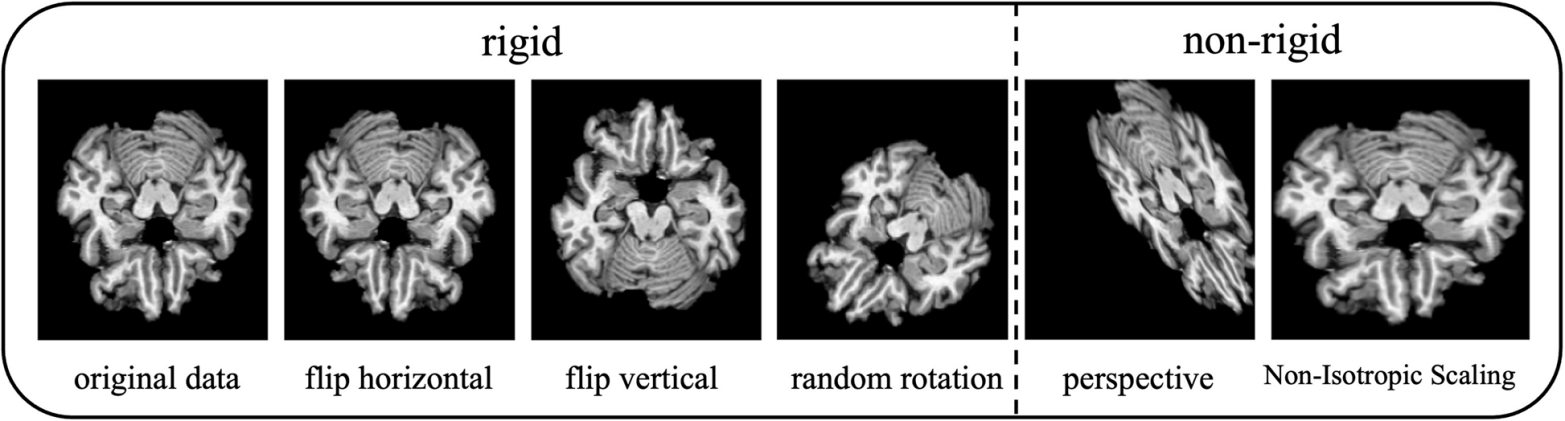
Some registration schemes involved in our study

In data preprocessing, rigid transformations include random rotation, and flip; non-rigid transformations include perspective, and Non-Isotropic Scaling. We show these methods in Fig. 1.

Computers store images in digital form, and each of these pixels is represented as a positive value. So we applied min-max normalization to the data, as shown in Eq. 1.

1 $$\begin{aligned} x^{'} = \frac{x - x_{min}}{x_{max} - x_{min}} \end{aligned}$$

But in image data, its minimum value is zero, so it can be calculated with Eq. 2.

2 $$\begin{aligned} x^{'} = \frac{x}{x_{max}} \end{aligned}$$

### Model design

**Fig. 2:**
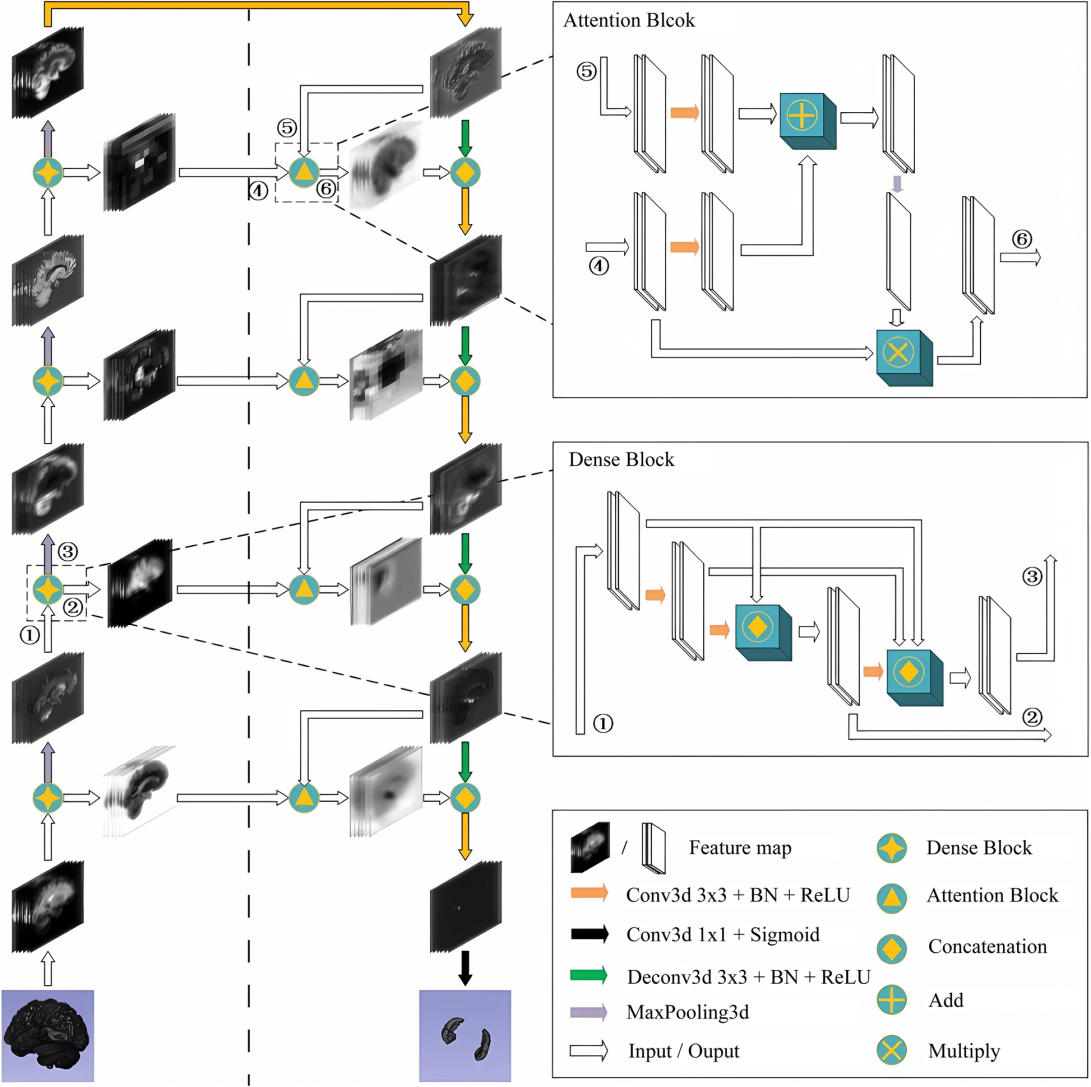
The overall architecture of the DeepHipp model. The model contains an encoding part and a decoding part. The encoder is on the left side of the auxiliary line, and the decoder is on the right side. In the encoding section, each module consists of a dense block. In the decoding section, each module consists of an attention block. The model uses end-to-end input and output to segment the hippocampus from the whole brain

We propose a 3D convolution model based on Dense block and attention mechanism. The input of the model is the complete brain data after preprocessing and augmentation. The topological structure of the DeepHipp network is shown in Fig. 2.

It consists of two parts: encoding and decoding. First, we look at the encoding part, the encoding part is composed of several dense blocks. Different from GoogleNet [29], ResNet [30], and other networks, these networks only consider the characteristics of adjacent layers. The characteristics of each layer are used up to once in the entire network, and the reusing rate of features is low, which makes the learning efficiency difficult enhancement. To improve the efficiency of feature usage, we reuse features of each layer learned by the network. The input of each dense block includes the image features by the preceding dense blocks so that the original features of the images are retained to the greatest extent. Secondly, let’s look at the decoding part. To capture the context information of the larger receptive field and semantic segmentation, the decoding part consists of several attention blocks. The original decoding layer is simply to concatenate the features of the encoding layer. However, the improved decoding layer uses the attention module to process the feature maps and concat the encoding layer. In other words, each attention block is connected to the corresponding encoding layer via a skip connection. Through the attention block, DeepHipp focuses on the segmentation target and suppresses irrelevant regions of the input image.

**Fig. 3:**
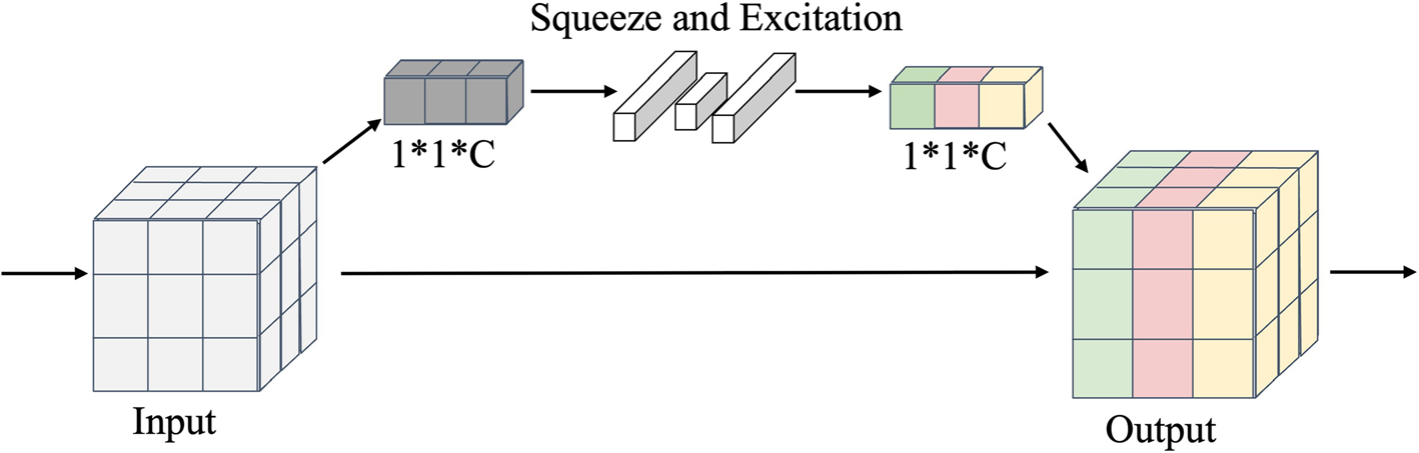
The structure of SE-layer

DeepHipp uses SE-layer, an attention mechanism that dynamically modulates the importance of different channels in the feature map for convolutional neural networks (CNNs). The structure of SE-layer is relatively simple, using global pooling or convolution layer to Squeeze the feature map as the weight vector of different channels. Then take Squeeze and Excitation operations on vectors, and automatically learn the weights of different channels during training, achieving the calculation of attention in channel dimensions. In the Squeeze and Excitation operations, we usually use a dense layer or a convolutional layer(kernel size is 1). Assuming that the reduction ratio is *r* and the size of the weight vector is *N*, the length of the weight vector changes as Eq. 3. Finally, the channel feature map is multiplied by the weight vector. The structure of SE-layer is shown in Fig. 3.

3 $$\begin{aligned} \left. N\rightarrow \frac{N}{r}\rightarrow N \right. \end{aligned}$$

In the network structure, except for the sigmoid activation function used in the output layer, all other activation functions are ReLU. Using sigmoid to normalize the results to (0,1), makes it easy to calculate the segmentation results at the output layer and determine the categories of each element in the matrix. The formula is in Eq. 4:

4 $$\begin{aligned} f(x) = \frac{1}{1 + e^{- x}} \end{aligned}$$

ReLU solves the problem of high computational complexity and easy gradient vanishing in sigmoid. ReLU will make the output of some neurons zero, making the network more sparser and alleviating overfitting. The formula is in Eq. 5:

5 $$\begin{aligned} f(x) = max(0,x) \end{aligned}$$

**Fig. 4:**
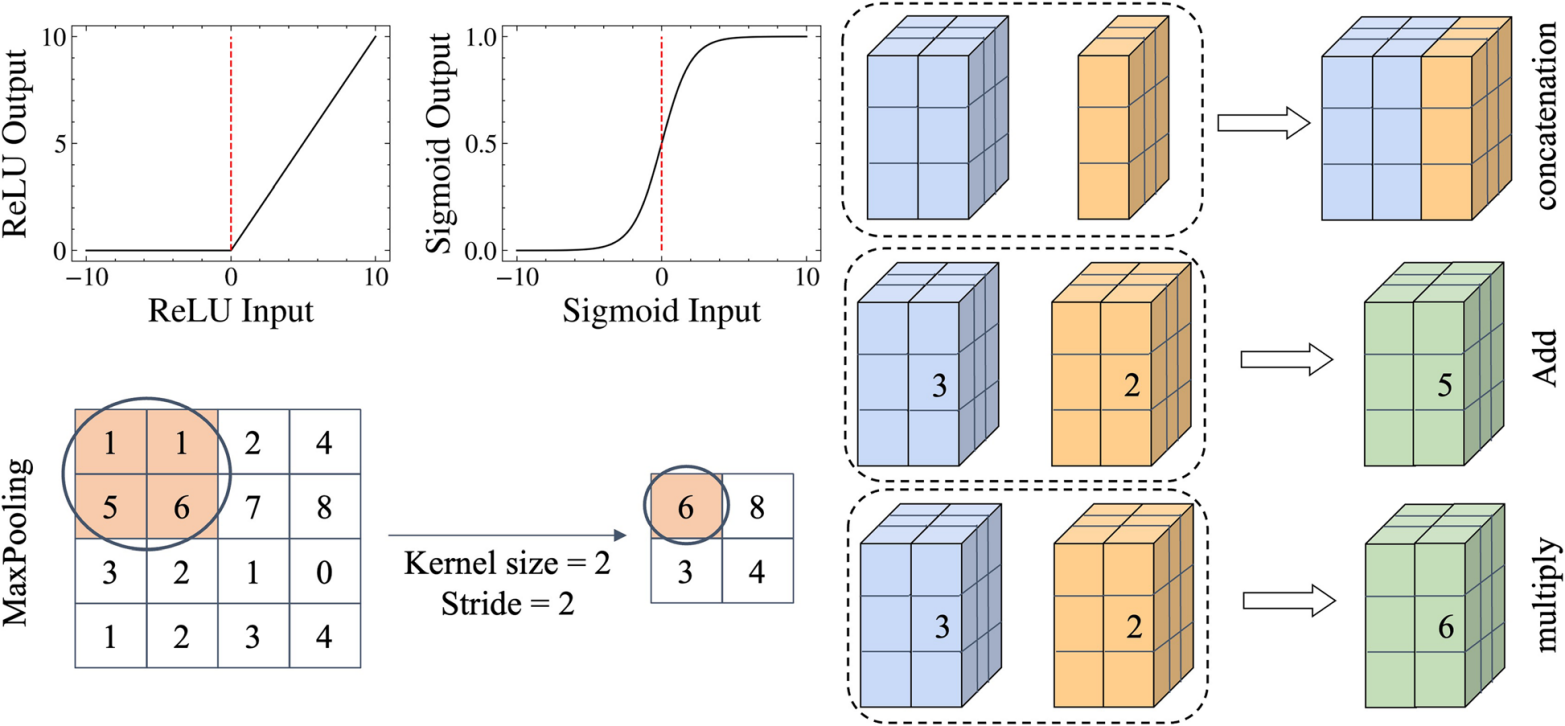
The mathematical formula for ReLU, sigmoid. And the processes diagram for concatenation, add, and multiply

During the downsampling process, we used maxpool3d, which can reduce the dimensionality of the feature map and accelerate the computational speed. This is a simple feature selection function that selects the maximum value in the target matrix as the output. As for the basic operations of tensors in neural networks, this study applies concatenation, add, and multiply. The above operation is shown in Fig. 4.

For the image classification problem, we often use cross-entropy as the loss function, as shown in Eq. 6.

6 $$\begin{aligned} CE\left( {y,~\overset{\sim }{y}} \right) = ~ - {\sum \limits _{i = 1}^{n}{y_{i}log\overset{\sim }{y_{i}}}} \end{aligned}$$

Where $y_{i}$ represents the true value, $~\overset{\sim }{y_{i}}$ represents the predicted value of the network, and n represents the number of pixels. However, for the segmentation problem, the traditional cross-entropy loss function does not work well. The current segmentation task often uses the Dice similarity coefficient as an objective criterion for segmentation accuracy, which is defined as Eq. 7.

7 $$\begin{aligned} Dice\left( {A,~B} \right) = ~\frac{2\left( {A \cap B} \right) }{A + B} \end{aligned}$$

A and B represent the real hippocampus region and the segmented region respectively of DeepHipp, representing the intersection of two regions. The dice similarity coefficient cannot be used as a loss function because it cannot propagate the network output loss backward. In recent years, many scholars have improved the Dice coefficient such as IoU [31] and Dice loss function [32]. The loss function used by DeepHipp is Eq. 8.

8 $$\begin{aligned} Dice\bigl ( y,~\overset{\sim }{y}\ \bigr ) = \frac{2{\sum \limits _{i = 1}^{n}{y_{i}{\overset{\sim }{y}}_{i}}}}{\sum \limits _{i = 1}^{n}\left( {y_{i}^{2} + {\overset{\sim }{y}}_{i}^{2}} \right) }~~ \end{aligned}$$

In the Results section, we show the impact of the hippocampus segmented by different loss functions for DeepHipp.

### Transfer learning

To achieve a better segmentation effect of the network, we use the transfer learning method to pre-train the network on the public datasets (BraTS). BraTS has accumulated a large number of data since 2015, and the goal of this competition is to encourage the development of state-of-the-art methods for tumor segmentation by providing a large dataset of annotated low-grade gliomas (LGG) and high-grade glioblastomas (HGG). The segmentation target of BraTS is the precise segmentation of three types of affiliated tumor regions. This segmentation task is obviously different from the natural image segmentation task with fewer categories. It needs the network to have the ability of precise segmentation for each kind of pathological tissue. By training the BraTS data, the network can achieve better convergence ability, and the pre-trained network weight parameters are migrated to the DeepHipp, which saves a lot of time and achieves higher precision for our target.

### Training and validating steps

We used 1000 original ADNI data and a preprocessing of the data in 2.1 to obtain approximately 3,000 samples. We use Keras (a kind of deep learning framework) to build the DeepHipp network. The initial batch size is set to 16, and the convolution kernel size is set to 3. The initial learning rate is set to 0.01, and the learning rate decreases as the iterations. All data is run under Linux. We use 6 Tesla-P100 GPUs. The normalized brain area is 160*160*192, and the number of convolution kernels increases with the network layers. We use parallel computing to speed up the processing of images [33]. Other DeepHipp’s parameters can be defined by users according to actual needs. Table 1 depicts a synoptic view of The DeepHipp segmentation process. $\chi$ represents sample space and epoch represents the number of iterations.

**Table 1:** Algorithm Description of DeepHipp

Algorithm Description of DeepHipp
Input: Training data with augmentation: $\chi$
1: Initialization: learning rate=0.01, bach_size=16, kernel size=3, randomly initialize *W*, *b*;
2: for $$epoch=1$$; $$epoch \le {epoch}_{max}$$; $$epoch++ do$$;
3: Compute $$Dice\left( {y_{i},~\overset{\sim }{y_{i}}} \right)$$;
4: Compute $$Loss = 1 - Dice\left( {y_{i},~\overset{\sim }{y_{i}}} \right)$$;
5: Compute $$Minimum\left( {Loss} \right) ;$$
6: Update the model parameter *W*, *b* based on $\chi$;
7: end for;
Output: The segmentation results from Model

## Results

**Fig. 5:**
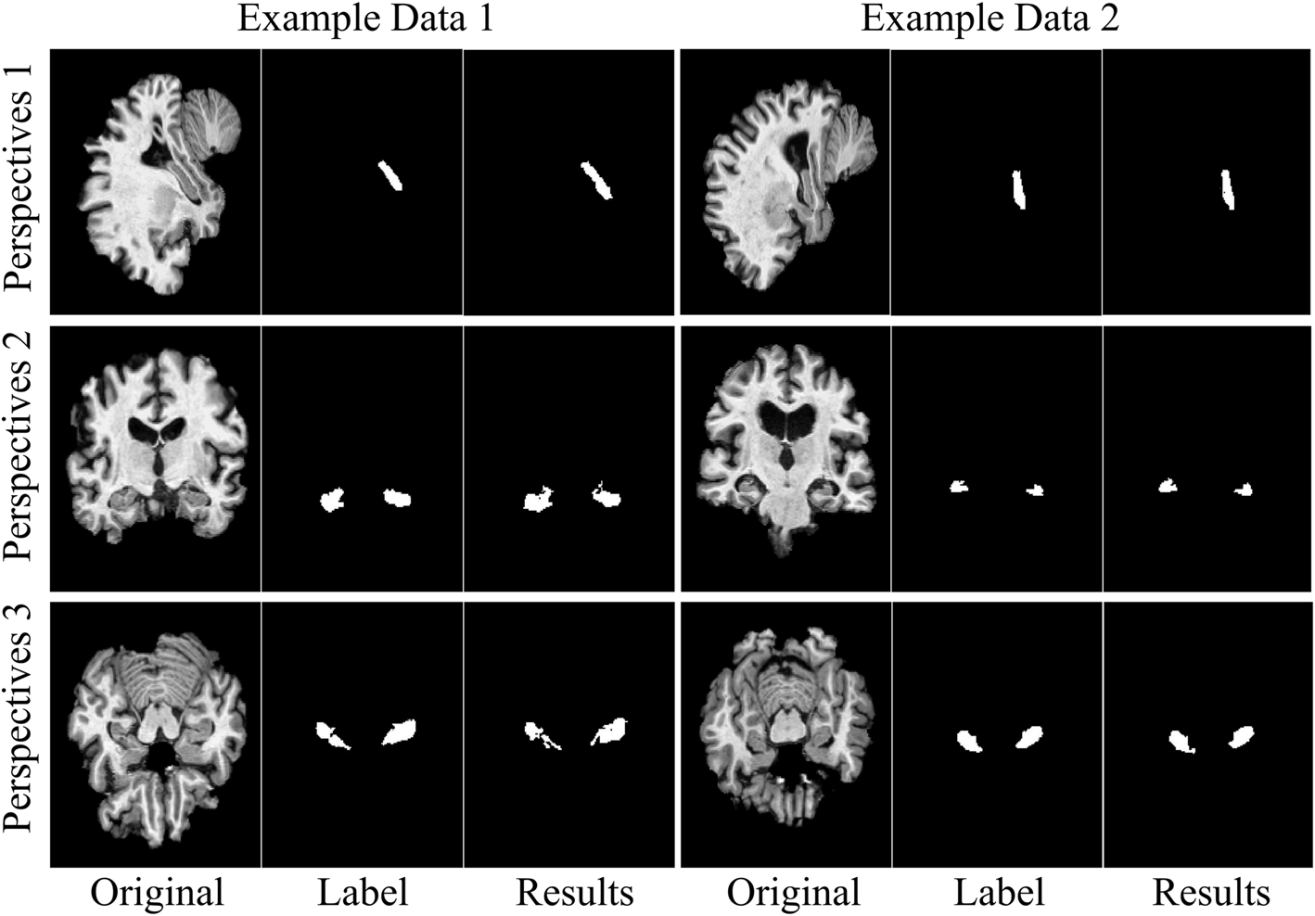
The visualization of original images, ground truth, and segmentation results of two data from three perspectives

DeepHipp can accurately segment the hippocampus region. However, due to the poor readability of the 3D image display, we visualized the original image, ground truth, and segmentation results from three perspectives. Individual data may have randomness, so we randomly selected two data for visualization, as shown in Fig. 5. To compare the overall accuracy of segmentation, we now validate the performance of DeepHipp using brain data from the ADNI Project. The training data set and the test data set are divided according to the following two criteria: (i) ensuring that the training data is sufficient for the model to converge. (ii) ensuring that the test data is sufficient to cover various targets to be detected. Under the premise of satisfying the above two criteria, the ratio of the training set and the test set can be adjusted according to the actual situation. In this experiment, the training set and the test set are 80% and 20% respectively. We compare DeepHipp with other mainstream segmentation network including FCN [34], Unet_3D [35], SegNet [36], PSPNet [37]. We examine various aspects of hippocampus’ segmentation by DeepHipp, including dice coefficient distribution, volume estimation, feature maps presentation, the effect of different models, the comparison with FreeSurfer.

### Dice distribution

**Fig. 6:**
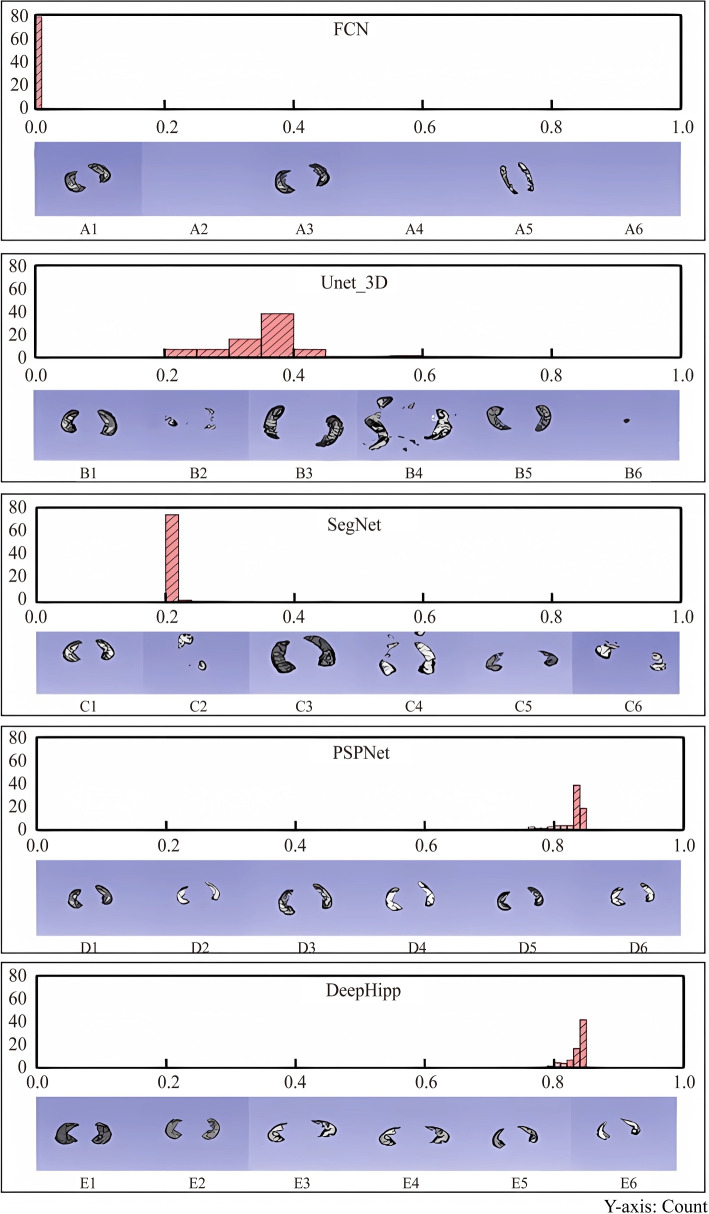
The histogram of the Dice coefficient. DeepHipp compared the Dice coefficients with the other four networks. Under each histogram, we take three groups of hippocampal segmentation samples, which are original data, geometrically transformation data, and resampling data. The graph on the left of each group is the label, and the graph on the right is the segmentation result

Firstly, we evaluate the dice coefficient distribution of DeepHipp and other segmentation models. We use the 80 brain samples from ADNI as the benchmark. We illustrate the results of the segmentation of the hippocampus with three data augmentation schemes. Figure 6 shows the histogram of different models’ dice distribution and examples of segmentation results.

To measure the performance of segmentation, we have counted the dice coefficient histograms of 80 individuals. At the bottom of each histogram is a comparison between the ground truth mask and the prediction. The first two pictures represent the comparison of the original data. The middle two pictures represent the comparison of geometric transformation. The last two pictures represent the data comparison after resampling. From Fig. 6, we can see FCN hardly recognizes the hippocampus whatever augmentation scheme. Unet_3D can slightly segment some brain regions, but it cannot clearly segment the outline of the hippocampus. SegNet can capture the contour of the hippocampus. PSPNet can segment the hippocampus completely, but the dice coefficient has not reached the optimal level. However, DeepHipp can segment the hippocampus completely, and the dice coefficient reaches a high level.

### Hippocampal volume

**Fig. 7:**
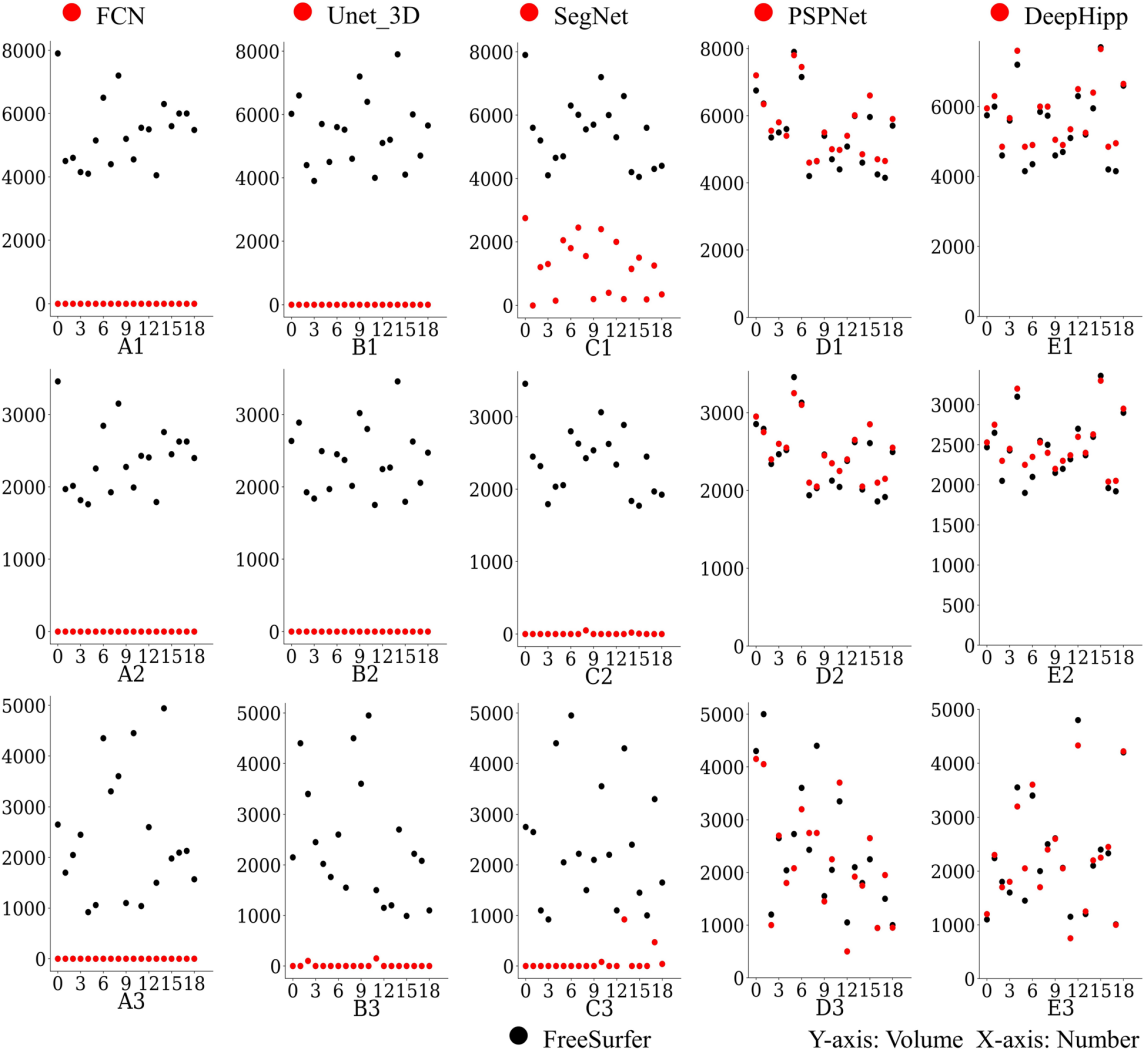
Hippocampus volume. The first line in Fig. 7 represents the segmentation results on the original data, the second line represents the segmentation results on the geometric transformation data, and the third line represents the segmentation results on the resampling data

As we all know, hippocampal volume is the basis for a variety of diagnostic tests. In this section, we further compare the hippocampal volume of DeepHipp with other segmentation models. We use the FreeSurfer segmentation as the standard reference. We randomly selected 20 individuals as the metrics and calculated the hippocampus volume of each individual to generate scatter plots, as shown in Fig. 7.

Each column in Fig. 7 represents a comparison of the different augmented schemes with each network and FreeSurfer. We can see that the volume of each segmentation result after the FCN network is empty, no matter with which data augmentation scheme FCN cannot segment any target. Similarly, Unet_3D has poor segmentation accuracy. For SegNet, under the original data, SegNet can segment part of the results, but the volume is much lower than the standard hippocampal volume. In the other two data augmentation schemes, the target area cannot be recognized at all. Further, we find that PSPNet and DeepHipp have better segmentation results under three data augmentation schemes. Under the original data, the two kinds of network segmentation results are similar. From the geometric transformation data, we can see that PSPNet results are slightly better than DenseNet, probably because PSPNet has some advantages over simple volume scaling. In resample data, we can see that DeepHipp results are better than PSPNet. In Fig. 7 E_3, blue and red dots coincide more. We analyze that DeepHipp plays an advantage in capturing the texture of the hippocampus and accurately segmenting the hippocampus when large brain deformation occurs.

### Feature maps visualization

**Fig. 8:**
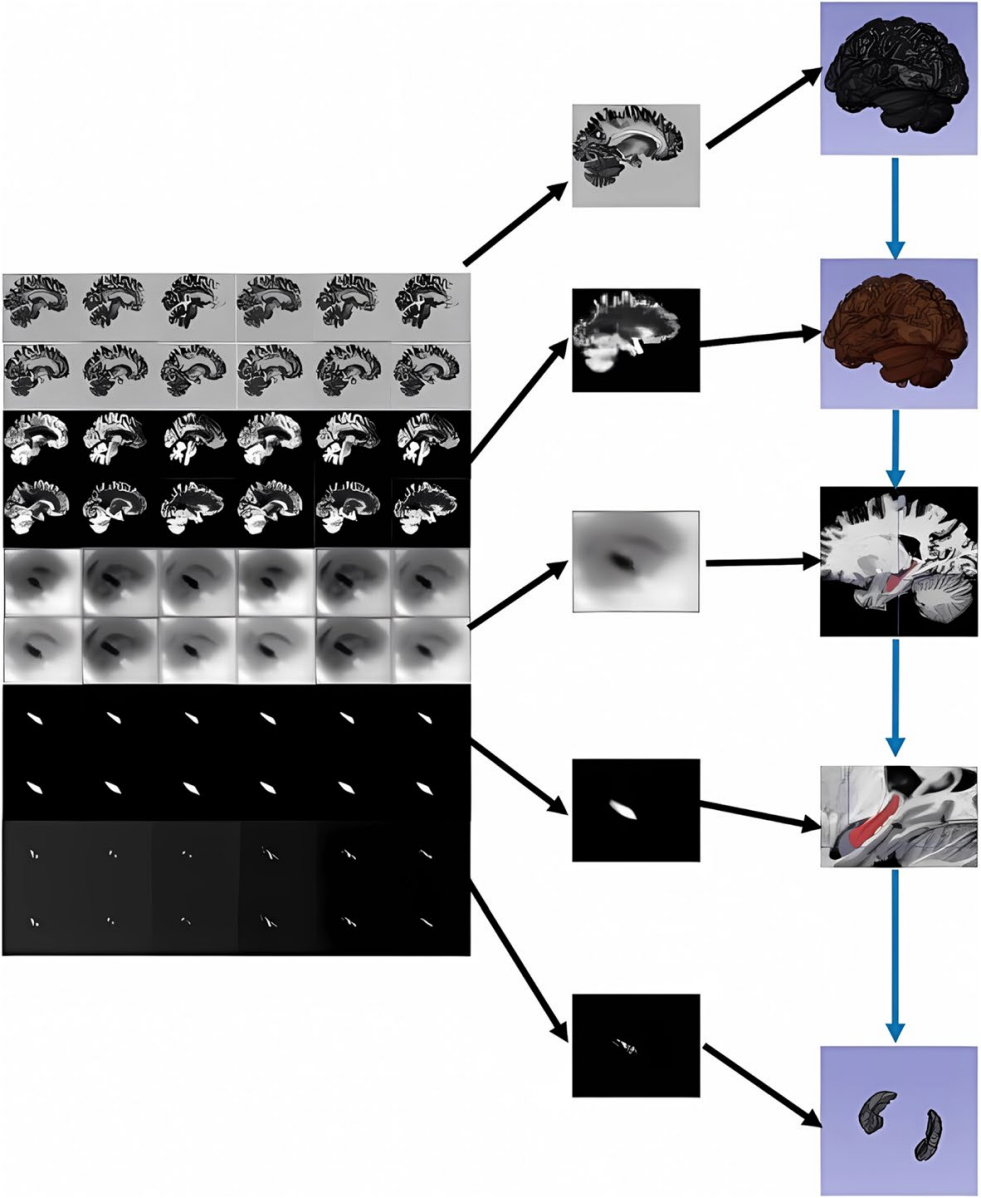
Feature Map display. We take the feature maps of representative network layers from top to bottom

In this section, we want to understand how the network captures the details of the hippocampus. We selected a representative network layer to display the feature maps. Since the 3D convolution used cannot better display the feature map, we take the slices after the convolution result to show. For each features cube, we select 12 slices from typical layers and organize the slices together for observation. We focused on the network’s dense-block and attention-block. As shown in Fig. 8, dense-block can reduce the vanishing gradient problem, and attention-block can make the network segmentation result more targeted.

In Fig. 8 left part, the first two lines are the feature maps of the first layer of DeepHipp. The second two line is the feature maps of the dense block. The third two lines are the feature maps of the attention block and the fourth two lines are the feature maps after the attention. The last two lines are the feature maps of the output. It can be seen that by using the dense-block under a limited amount of data, the network has a good anti-overfitting effect. At the same time, the advantage of using attention-block is that the network can scan the global image quickly, and then invest more attention in this area to get more details of the target, to suppress other useless information.

### Performance of Multiple Models

**Fig. 9:**
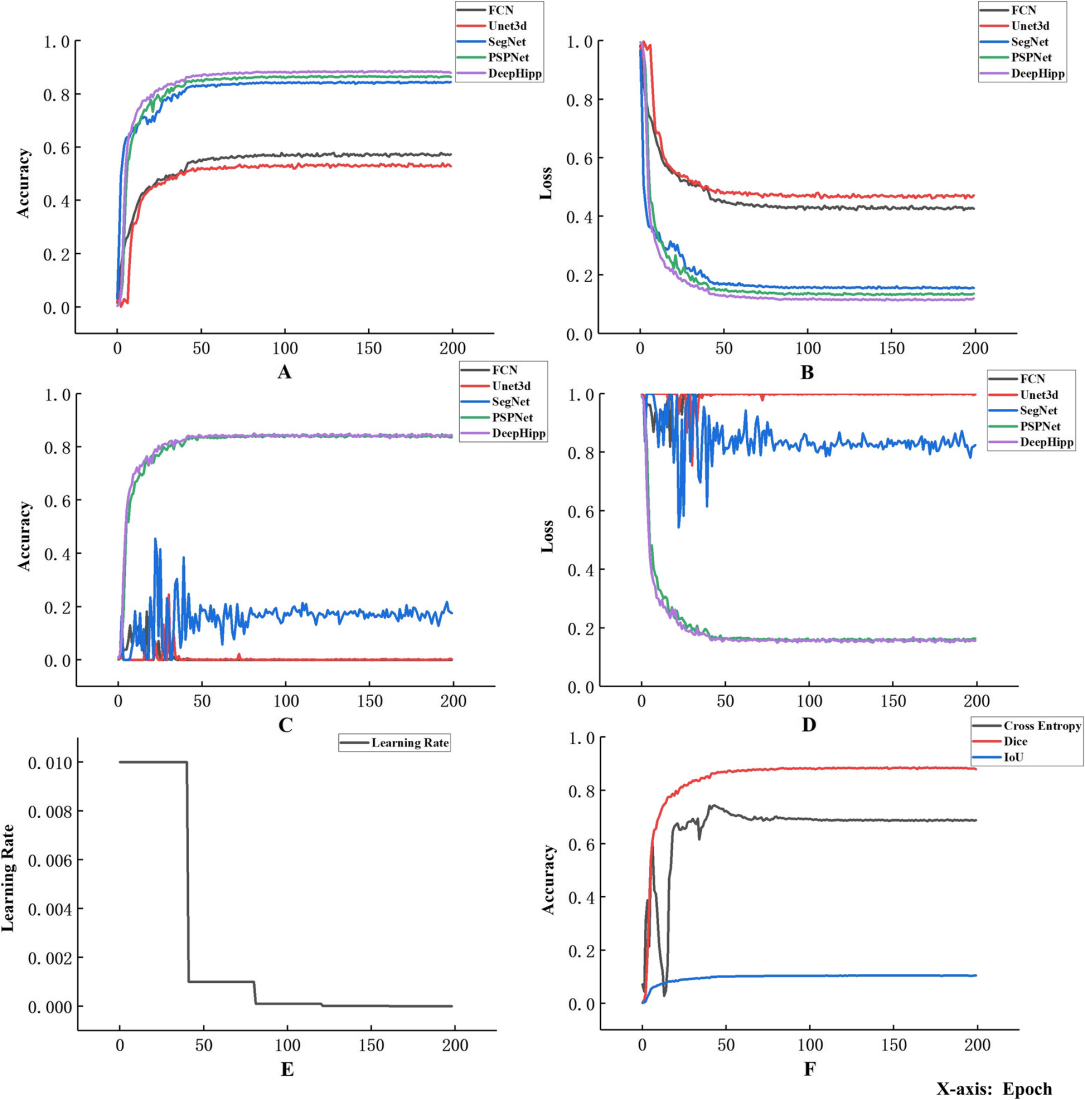
Performance of Multiple Models. **A** and **B** represent the accuracy and loss of the different segmentation networks on the training set. **C** and **D** represent the accuracy and loss of the different segmentation networks on the validation set. In the four graphs **A**, **B**, **C**, and **D**, we can infer that DeepHipp outperforms other algorithms. During the testing process, FCN and Unet_3D accuracy is almost zero. The results of SegNet are unstable, approximately 0.2. PSPNet and DeepHipp can reach 0.8315 and 0.8363 respectively. Graph **E** represents the change in learning rate during the training process. **F** shows the training accuracy using different loss functions of DeepHipp. Using the dice coefficient can achieve the best results

To verify the advantages of DeepHipp with other existing medical image segmentation networks, we compare the performance of DeepHipp and FCN, Unet_3D, SegNet, and PSPNet from various aspects, including the accuracy, loss, and learning rate in the training set and validation set, as shown in Fig. 9.

We know that for the semantic segmentation model, FCN can be regarded as the originator. It is the infrastructure of the segmentation model in many fields. We apply the training data to the FCN model, but the experimental results are not satisfying. Similarly, Unet models have unique advantages in the field of medical imaging. For 3D unet, our training results show that there is still no high accuracy. For the SegNet proposed in recent years, we find that it achieves relatively good results in the training set, but there is no high accuracy and low loss in the validation set. The reason for this may be that SegNet transfers the maximum pooling into the decoder, which improves the segmentation resolution but fails to meet the expected accuracy requirements. For PSPNet and DeepHipp, both the training set and the validation set have achieved high accuracy, but as mentioned in Dice distribution section, the poor robustness of PSPNet leads to the decrease of segmentation precision in the case of brain deformation, while DeepHipp has strong adaptability and can still accurately segment the hippocampus in the case of brain deformation. Finally, we verify the training accuracy for DeepHipp using different loss functions. We can see Dice coefficient is the best of all loss functions.

### Comparison with FreeSurfer

**Fig. 10:**
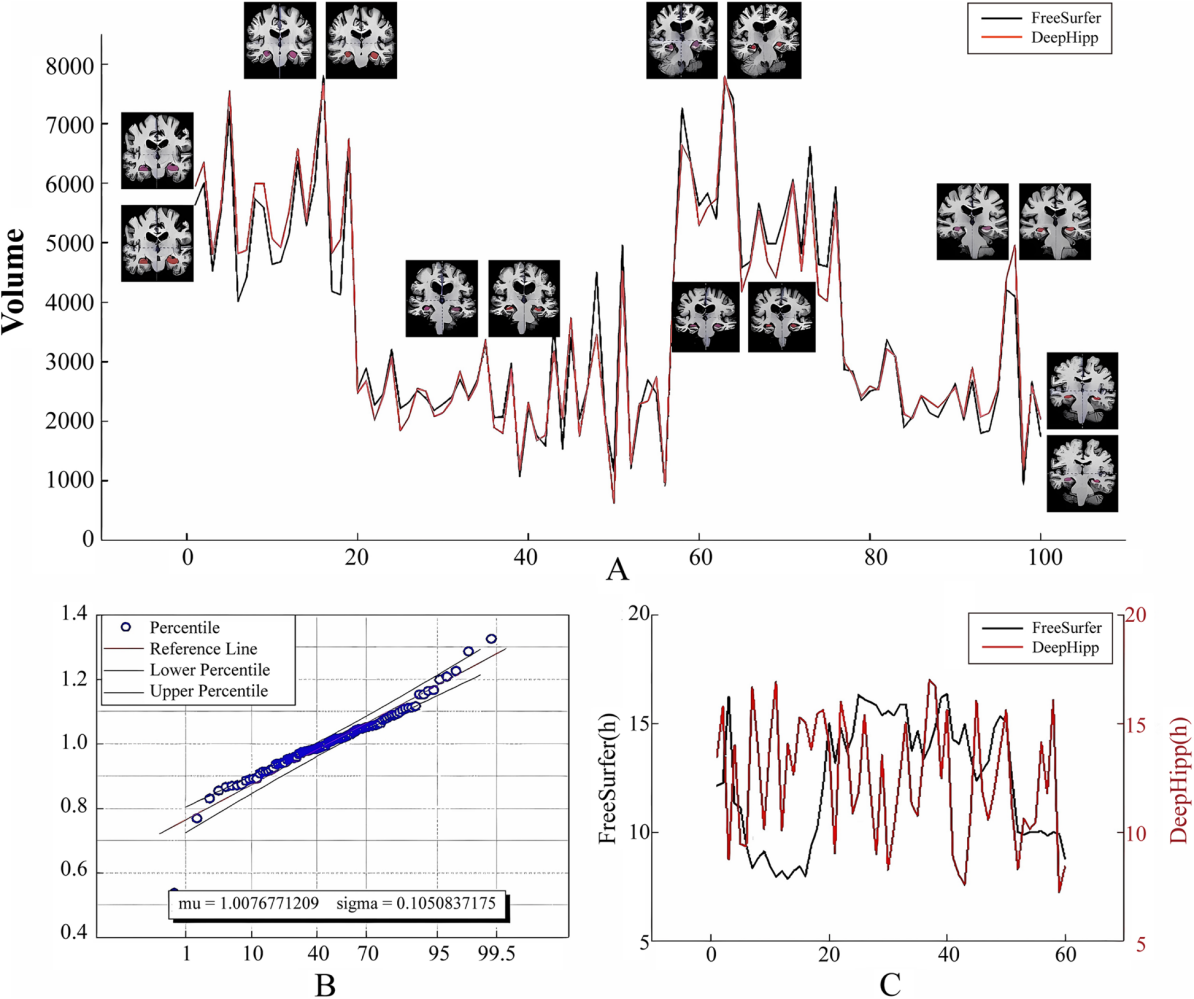
DeepHipp vs. FreeSurfer. **A** shows the segmentation results for 100 samples and shows a comparison of FreeSurfer and DeepHipp’s MRI at different peaks. **B** shows the division of the hippocampus volume by FreeSurfer and DeepHipp and counts the mean and variance values. **C** shows the segmentation time for FreeSurfer and DeepHipp segmentation. Note that FreeSurfer segments the hippocampus in hours but DeepHipp does in seconds

In the last section, to see whether DeepHipp performs consistently on other ADNI data, DeepHipp chooses 100 individuals to verify the precision. And these 100 individuals are not included in the training set and test set. Note that, we sent the same data to FreeSurfer, and made volume statistics on the segmented results. The relevant statistical results are shown in Fig. 10.

As shown in Fig. 10-A, we selected a representative group of examples to compare FreeSurfer and DeepHipp segmentation. However, we found that in subjects with hippocampal atrophy, FreeSurfer segmentation results were slightly larger than the actual hippocampal, while DeepHipp was well consistent with the actual hippocampal. The analysis of these reasons may be that Freesurfer is a template-based comparison algorithm, but DeepHipp is a data-driven learning model. We counted the voxel points of 100 hippocampus separated by FreeSurfer and DeepHipp and made a polyline map. We can see that the red line and the black line have a high consistency. To further show the segmentation ability of DeepHipp, we divide the hippocampal volume of Freesurfer by the hippocampal volume of DeepHipp. We then calculated the mean value and variance of 100 points. It can be seen from Fig. 10-B that the mean value is close to 1, and the variance is close to 0.1, indicating that the hippocampus segmented by DeepHipp is very similar to FreeSurfer. Finally, we also compare the segmenting time required for the DeepHipp network and the time required for FreeSurfer in Fig. 10-C. It is found that DeepHipp greatly shortens the hippocampus segmentation period.

## Discussions

In this paper, we present a precise hippocampus segmentation network using T1 data from ADNI. Firstly, we make a novel and complex augmentation scheme for ADNI data. To make the network more effective, we expand the original data in shape, contrast, and data magnitude. Secondly, in the construction of the deep learning model, we adopt the latest attention mechanism based on computer vision, which greatly improves the recognition of the target segmentation area. At the same time, to improve the inhibition of gradient disappearance, we add the dense-block module, which improves the learning ability and successfully avoids over-fitting. Finally, we compare DeepHipp with the existing mainstream segmentation networks. The results show that DeepHipp has high accuracy and sensitivity in hippocampal segmentation.

In the past decade, with the rapid development of computer vision, medical imaging equipment has been constantly updated and iterated. From the previous single T1 data to the present T2 data, the imaging resolution is increasing day by day. The hospital produces thousands of images every day. Such a large scale of data lays a good foundation for the application of deep learning in this field. Using a deep learning network, DeepHipp can learn the regularity and characteristics of massive data to achieve the purpose of assisting medical diagnosis. Reviewing the existing methods of hippocampal segmentation, most of them are based on template and morphological methods, such as FSL, and FreeSurfer. Their common feature is that the detection time is too long to segment the target. For example, it takes at least 6 hours to segment a hippocampus in FreeSurfer and 20 minutes to perform the same operation on FSL. However, it only takes 20 seconds to detect a hippocampus in DeepHipp, which greatly improves the efficiency of doctors and reduces the workload. Through our experiments, for a single data, only 2GB of GPU memory is occupied during the inference stage.

The DeepHipp proposed in this paper, for the characteristics of medical data three-dimensional imaging, does not use the traditional 2D convolution but uses the form of 3D convolution. DeepHipp can capture the details of objects very well in three-dimensional medical images and can find out the differences among voxel points. Because medical data itself is rarely labeled, unlike natural images, which have a large number of manual labels, medical image labeling requires a lot of human and material resources. This requires us to train a segmentation model with higher generalization ability under a relatively small dataset. DeepHipp uses the dense-block to avoid the vanishing gradient problem caused by larger parameters of the network and to resist the over-fitting effect caused by a smaller dataset. In recent years, the attention mechanism has been widely used in natural language processing and image recognition. DeepHipp has successfully added an attention mechanism in the network construction, which greatly improves the precision of segmentation. DeepHipp can focus its attention on the hippocampus from a large amount of training data. We can observe the advantages of the attention mechanism from the feature map.

Finally, DeepHipp is currently only a detection tool for hippocampal segmentation. In the future, we hope that DeepHipp can be used to detect more brain areas, such as the frontal lobe, white matter, and gray matter. At the same time, we only use the result of Freesurfer segmentation as DeepHipp training masks. In the future, we can integrate more masks as DeepHipp training labels. If conditions allow, we can increase a large number of manual labels as DeepHipp to improve the segmentation ability of DeepHipp. Furthermore, since medical image data are multi-modal, we hope that DeepHipp can detect not only single modal data but also multiple modalities in the future.

## Conclusions

In this paper, we elaborate on revealing a novel method for segmenting the hippocampus. To solve this, we propose a new segment model using a 3D dense-block based on an attention mechanism, named DeepHipp. Unlike the previous conventional tools, DeppHipp can extract meaningful knowledge from a succession of brain samples. Above all, DeepHipp has good capability at feature recognition and mechanism of target attention, and can quickly separate the hippocampus from the brain. We believe that deep learning can potentially lead to effective segmentation of medical imaging and be applied to many other medical questions.

## Data Availability

We use the real data from ADNI Project at https://adni.loni.usc.edu. The software and sample result as part of this project are readily available from GitHub at https://github.com/CSuperlei/DeepHipp.

## Abbreviations

MRI: Magnetic resonance imaging
PTSD: Posttraumatic Stress Disorder
FSL: FMRIB’s Software Library
ANTs: Advanced Normalization Tools
CNN: Convolution Neural Network
BraTS: Brain Tumor Segmentation with Deep Neural Networks
MoE: Sparsely-Gated Mixture-of-Experts layer
GoogleNet: Google Inception Net
ResNet: Deep residual network
IoU: Intersection over Union
LGG: low-grade gliomas
HGG: high-grade glioblastomas
ADNI: Alzheimer’s Disease NeuroImaging Initiative
FCN: Fully Convolutional Networks
PSPNet: Pyramid Scene Parsing Network
